# Linking Brain Arteriovenous Malformations With Anorectal Hemorrhoids: A Clinical and Anatomical Review

**DOI:** 10.1002/ar.23643

**Published:** 2017-07-21

**Authors:** Joshua A. Cuoco, Christopher L. Hoehmann, Kyle Hitscherich, Sherry M. Zakhary, Joerg R. Leheste, German Torres

**Affiliations:** ^1^ Department of Biomedical Sciences New York Institute of Technology College of Osteopathic Medicine Old Westbury New York; ^2^ Department of Anatomy New York Institute of Technology College of Osteopathic Medicine Old Westbury New York; ^3^ Department of Radiology Brookhaven Memorial Hospital Medical Center Patchogue New York

**Keywords:** vascular dysmorphogenesis, hereditary hemorrhagic telangiectasia, intracranial hemorrhage, magnetic resonance imaging, nutritional substances

## Abstract

Patients who harbor brain arteriovenous malformations are at risk for intracranial hemorrhage. These malformations are often seen in inherited vascular diseases such as hereditary hemorrhagic telangiectasia. However, malformations within the brain also sporadically occur without a hereditary‐coding component. Here, we review recent insights into the pathophysiology of arteriovenous malformations, in particular, certain signaling pathways that might underlie endothelial cell pathology. To better interpret the origins, determinants and consequences of brain arteriovenous malformations, we present a clinical case to illustrate the phenotypic landscape of the disease. We also propose that brain arteriovenous malformations might share certain signaling dimensions with those of anorectal hemorrhoids. This working hypothesis provides casual anchors from which to understand vascular diseases characterized by arteriovenous lesions with a hemorrhagic‐ or bleeding‐risk component. Anat Rec, 2017. © The Authors. The Anatomical Record published by Wiley Periodicals, Inc. on behalf of American Association of Anatomists. Anat Rec, 300:1973–1980, 2017. © 2017 The Authors. The Anatomical Record published by Wiley Periodicals, Inc. on behalf of American Association of Anatomists.

The anatomical architecture of brain arteriovenous malformations (BAVMs) consists of tangles of aberrant arterial and venous vessels in the brain (Fig. 1A,B). The clustering mass of blood vessels that emerge from this particular vascular anomaly is known as a nidus, an endothelial lesion which often presents with intracranial hemorrhage (ICH). Though relatively uncommon (<1% in the general population), BAVMs represent a significant cause of morbidity due to the high incidence of ICH (Fullerton et al., 2005; Friedlander, 2007; Moht et al., 2013).

**Figure 1:**
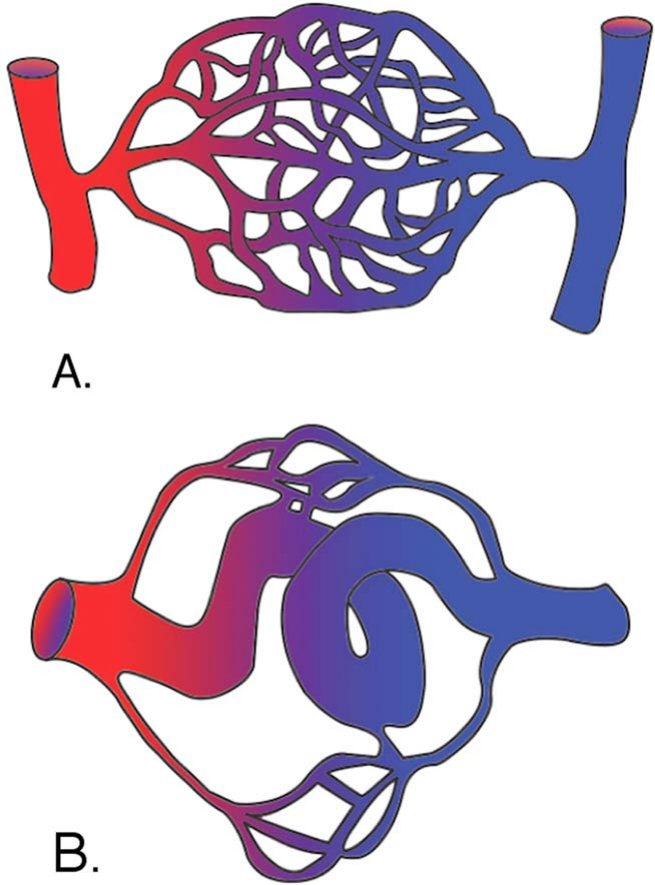
Schematic diagrams depict a wild‐type/normal (A) and a pathological arteriovenous system (B) that presents in the brain parenchyma of patients with BAVMs. This rare condition is the result of an aberrant connection between arterial and venous systems in the absence of intervening blood‐carrying capillaries. As a consequence, forced high‐pressure exerted by the arterial system weakens the thin endothelial walls of the venous system possibly through the breakdown of a proteoglycan matrix required specifically for the brain vasculature. This loosens gap junctions and precipitates ICH. It should be noted that BAVMs are rarely seen during routine prenatal sonography, thus preventing early diagnosis and experimental treatment of the disorder.

Currently, there are few therapeutic options that can be administered to control the neurological symptoms associated with BAVMs (Van Beijnum et al., 2007; Macellari et al., 2014). Furthermore, there are no FDA‐approved drugs that inhibit BAVM progression or impede the occurrence of ICH (Achrol et al., 2009). Thus, a better understanding of the molecular mechanisms of BAVMs is essential for designing novel therapies. Here, we review our current understanding of the pathogenic mechanisms of BAVMs, describe the clinical nosology of the syndrome and put forward a working hypothesis linking the formation of anorectal hemorrhoids to BAVMs.

## Pathogenesis and System Genetics

BAVMs are thought to arise between the 4th and 8th week of human embryonic development; however, there is a lack of strong evidence for this tentative hypothesis (Gross et al., 2012; Ieva et al., 2015). In the adult patient, the mean age of diagnosis is ∼40 years, but whether older age is associated with increased burden pathology remains to be explored (Gross et al., 2012). Contrary to other vascular pathologies such as arteriovenous fistulas or epidural hematomas, BAVMs are not associated with cranial trauma nor are they linked to any particular environmental risk factor (Gross et al., 2012; Ieva et al., 2015). The etiopathogenesis of BAVMs is therefore not fully understood. However, BAVMs are known to be associated with vascular diseases, such as hereditary hemorrhagic telangiectasia (HHT) and sporadic vascular malformations that cause hemorrhagic strokes and epileptic seizures in young patients (Gross et al., 2012; Ieva et al., 2015; Macellari et al., 2014; Zhou et al., 2016). The fact that most cases of BAVMs are sporadic (≥95%) rather than familial suggests (i) *de novo* coding mutations that arise in gametes, (ii) single‐nucleotide polymorphisms (SNPs) with low penetrance or (iii) rare protein‐disrupting mutations might contribute to disorders characterized by the abnormal aligning of endothelial cells. These suggestions blur not only BAVMs origins but also variability of syndromes between patients harboring different forms of somatic mutations. From this perspective, BAVMs are a collection of rare conditions with extensive behavioral heterogeneity and whose symptoms are resistant to specific therapies. This is particularly true for the brain's microvasculature, which is poorly understood at the molecular level, with numerous signaling pathways coordinating early endothelial cell growth, differentiation and proliferation (Zhou et al., 2015, 2016). Finally, it should be noted that at the whole‐genome scale, each patient has millions of native intronic variants (Baralle and Baralle, 2005). Many of these variants or haplotypes do not code for proteins but instead regulate gene activity, so they can still contribute to the formation of a high‐flow nidus.

**Figure 2:**
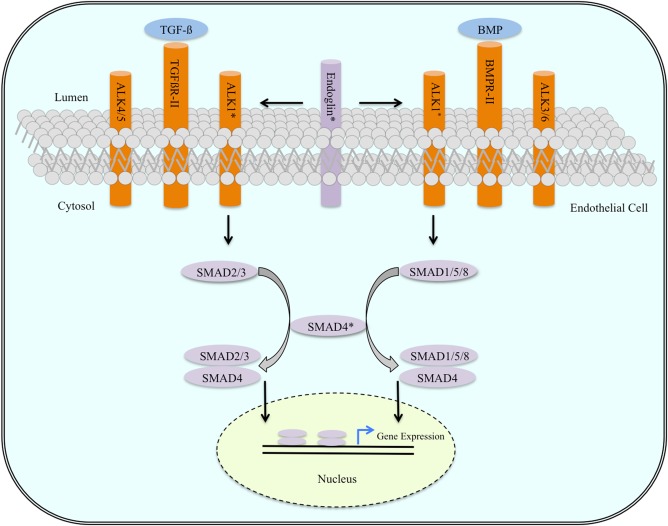
This schematic diagram depicts the signaling pathways that possibly drive BAVM pathology. In brief, TGF‐β or BMP binds to TGFβR‐II and BMPR‐II, respectively. Activated TGFβR‐II and BMPR‐II phosphorylate their respective type I receptor proteins. TGFβR‐II activates ALK‐1 and ALK‐4/5 whereas BMPR‐II activates ALK‐1 and ALK‐3/6. ALK‐1 will activate SMADs 2 and 3 as well as SMADs 1, 5, and 8 in their respective signaling pathways. Both SMAD complexes further associate with SMAD4, translocate to the nucleus and activate gene transcription contributing to BAVMs pathology. ENG is a coreceptor regulator of TGFßR‐II and BMPR‐II and attenuates the activity of both signaling pathways. Mutated ENG will result in constitutive activity of the TGF‐β signaling pathway downstream. *Indicates mutated proteins identified in hereditary hemorrhagic telangiectasia. Intracranial hemorrhage can be better understood by characterizing key molecular mechanisms responsible for the onset of BAVMs. No single genetic abnormality accounts for a large number of cases of the disease. Instead, many genes hit across different patients, each with a relatively small effect on ICH risk. Abbreviations: TGF‐β—transforming growth factor‐β; BMP—bone morphogenic protein; TGFβR‐II—transforming growth factor‐β receptor type II; BMPR‐II—bone morphogenic protein receptor type II; ALK—activin‐like kinase; SMAD—small body size/mothers against decapentaplegic.

Regardless of rates, spectrum and determinants of genomic mutations, BAVMs share common susceptibility loci with HHT. HHT, also known as Osler‐Weber‐Rendu syndrome, is an autosomal dominant disorder characterized by the presence of vascular malformations in the skin, lung, liver and brain (Gross et al., 2012; Moht et al., 2013; Goyal et al., 2014; Ieva et al., 2015). Two primary clinical subtypes of HHT are currently clustered. Both subtypes exhibit loss‐of‐function mutations in genes encoding segments of the transforming growth factor‐β (TGF‐ß) signaling pathway (Fig. 2). For instance, HHT‐1 exhibits mutations in endoglin (ENG), which codes for an accessory protein of the TGF‐β adaptor complex (Gross et al., 2012; Goyal et al., 2014; Sahlein et al., 2014; Ieva et al., 2015), whereas HHT‐2 presents with mutations in activin‐like kinase 1 (ALK‐1), which codes for a transmembrane‐bound kinase receptor associated with the transmission signature of TGF‐β (Gross et al., 2012; Goyal et al., 2014; Sahlein et al., 2014; Ieva et al., 2015). TGF‐β is a cytokine regulating critical developmental milestones such as cell differentiation, migration, adhesion and proliferation. Paradoxically, TGF‐β also promotes tumor formation, invasion, and metastasis in self‐renewing tissues (Abendstein et al., 2000; Abetov et al., 2015). Thus, for some forms of BAVMs, there is evidence for a heritable risk threshold with respect to expressivity and severity of disease. Knowing how TGF‐β signaling pathways reflect genetic risk at the level of the patient is necessary to develop a mechanistic understanding of BAVMs.

As discussed above, most forms of BAVMs are sporadic (e.g., those observed in a child but not the parents) reflected in patterns of SNPs expressivity within genes encoding pro‐inflammatory cytokines such as interleukin‐1 β (IL‐1 β), tumor necrosis factor‐ α (TNF‐α) and matrix‐metalloproteinase (MMPs). The findings that pro‐inflammatory cytokines might contribute to nidus formation in the brain do not yet deliver a complete picture of the genetic landscape of BAVMs. However, there is sufficient information to draw some general conclusions. For instance, pro‐inflammatory cytokines might intersect with transcription factors such as the forkhead box O (FOXO1) to adjust vascular density and vascular expansion during specific phases of endothelial cell sprouting (Wilheim et al., 2016). Indeed, if the activity of FOXO1 is restricted, at least in animal models, it leads to a coordinated reduction in blood vessel branching and blood vessel expansion in mutant retinas (Wilheim et al., 2016). Furthermore, cerebral cavernous malformations, which are also sporadic in expressivity, appear to arise from endothelial gain of MEKK3‐KLF2/4 signaling. Abnormally high levels of this adaptor complex cause specific vascular lesions in mouse and human brains; lesions which are characterized by hemorrhagic dilated venules in the white matter of the cerebellum (Zhou et al., 2016). Against this background, we can begin to formulate the hypothesis that the origin of BAVMs lies in *de novo* or inherited patterns of transmission, with numerous loss‐ and gain‐of‐function signaling pathways interacting broadly with networks of local endothelial molecules to produce a defective vascular phenotype.

## Clinical Case Presentation

**Figure 3:**
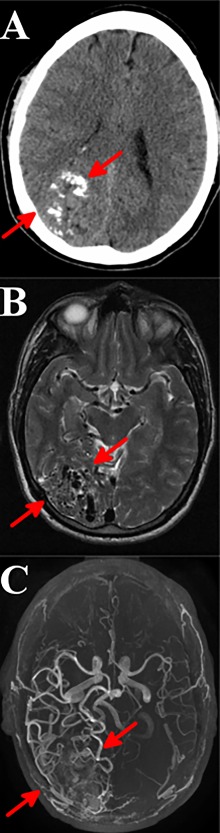
Computerized tomography (CT), MRI and angiography (MRA) axial images of a patient diagnosed with a BAVM. The initial diagnosis of BAVM is based on behavioral symptoms and on differentiation from other brain disorders. Multiple structural imaging techniques are often required to definitively diagnose the arteriovenous syndrome. Image (A; red arrows) depicts an unenhanced axial CT image at the level of the lateral ventricles demonstrating a cluster of serpiginous coarse calcifications in the right occipital lobe which is suggestive of vascular calcification. Image (B; red arrows) depicts an axial T2‐weighed MR image at the level of the midbrain demonstrating several prominent flow‐voids in the right occipital lobe which is suggestive of an arteriovenous malformation. Image (C; red arrows) depicts an unenhanced reconstructed axial time‐of‐flight maximum‐intensity projection image of the Circle of Willis demonstrating a right posterior arteriovenous malformation with feeding arteries from the right posterior cerebral artery and branches of the right middle cerebral artery.

Because the protein products of genes regulating endothelial cell growth, differentiation, and proliferation often interact with one another, the signaling pathways within them overlap at multiple levels of molecular, cellular, and circuit function. Therefore, it should not be surprising that different gene networks can yield the same clinical phenotype, as seen in BAVMs. Recognition of these signaling pathways has important ramifications for our ability to understand inter‐patient heterogeneity. A brief clinical case of BAVM illustrates (i) the heterogeneity of the syndromic presentation, (ii) the overall clinical picture of this rare brain disorder (Box 1) and (iii) the use of structural magnetic resonance imaging (MRI) to decode the surface morphology of arteriovenous lesions (Fig. 3A–C).

**Figure 4:**
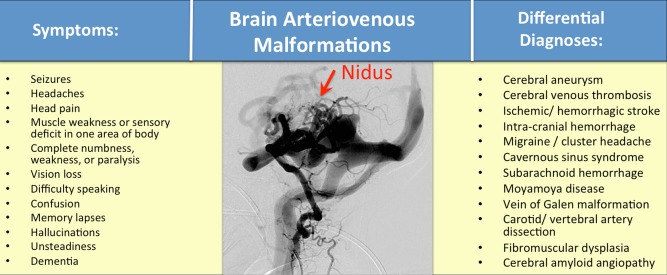
A representative BAVM with a central nidus (red arrow) is shown along with symptoms and differential diagnoses of the disease. The image above is a digital subtraction angiogram demonstrating a well circumscribed nidus and multiple feeding arteries and draining veins. A broad range of behavioral symptoms and structural brain processes can produce changes that mimic BAVM pathology. Note that BAVMs can produce measurable neurocognitive deficits. Traditional diagnostic tools as history, symptomatology and physical signs along with brain scans and possibly additional comorbidities (e.g., anorectal hemorrhoids) will help discriminate BAVMs from other brain disorders. In general, the differential diagnoses of BAVMs are broad, with often overlapping diagnostic features that can lead to diagnostic confusion. This increases individual and family burden, and causes patients to seek unhelpful therapies or join the wrong support groups.

Box 1 A 43‐year‐old male patient without previous clinical history first presented to the emergency department (ED) with a complaint of “passing out while driving,” giving the impression of a generalized epileptic seizure (Fig. 4A–C). MRI and CAT brain scans revealed a right occipital AVM. The patient returned to the ED at the age of 51, after having a near‐syncopal episode. Brain imaging scanning again confirmed the AVM diagnosed 8 years prior. As the patient had no surgical intervention for the BAVM lesions, the immediate treatment was focused on the management of his blood pressure to prevent vessel rupture and minimize the occurrence of ICH.

## Common Dimensions of Dysfunction between Brain Arteriovenous Malformations and Anorectal Hemorrhoids

**Figure 5:**
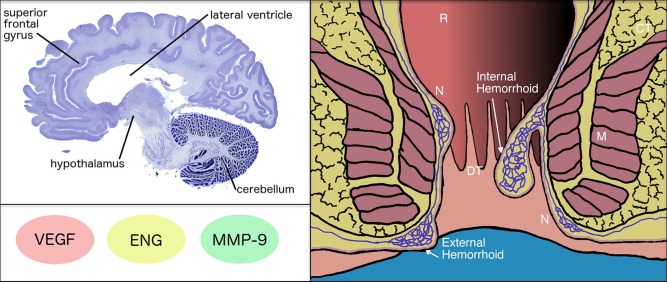
This schematic diagram depicts the clinical presentation of anorectal hemorrhoids and signaling dimensions (e.g., VEGF, ENG, and MMP‐9) shared with BAVMs. It is known that pathological remodeling of vascular structures within anorectal cushions can lead to hemorrhoids. More specifically, changes in the superior and inferior plexus of the dentate line can induce vascular hyperplasia and vessel enlargement. We came up with an interesting hypothesis, and possibly insights, into risk factors for brain disease by studying the anorectal tract. Basic principles of hemorrhoid biology might be applied to the study of brain disorders, such as BAVMs. It is not known whether there a comorbidity link between BAVMs and anorectal hemorrhoids. However, attempts to develop biomarkers for BAVMs may benefit from a hemorrhoid screening protocol to stratify patients for clinical testing of targeted drugs, including dietary molecules. Abbreviations: DT (dentate line), R (rectum), CT (connective tissue), M (muscle), N (normal cushion). Human brain*: sagittal plane; Nissl stain. For spatial orientation, several brain regions are labeled. Image from the Yakovlev‐Haleem collection is used courtesy of the National Museum of Health and Medicine, Armed Forces Institute of Pathology. *Michigan State University Brain Biodiversity Bank.

Blood vessels must sprout, elongate, form lumens and regress to adjust their growth state for normal endothelial redox function (Wilheim et al., 2016). The findings that certain loss‐ and gain‐of‐function mutations blunt the growth state of the vascular endothelium suggest that the aforementioned mutations might also contribute to similar dimensions of vascular dysfunction outside the brain parenchyma. One area of potential overlap involves the aberrant arteriovenous malformations (AVM) that characterize anorectal hemorrhoids (Sanchez and Chinn, 2011). Although it is not known whether BAVMs and anorectal hemorrhoids share substantial comorbidity or obvious etiology signature, it is interesting that both syndromes share causal signaling pathways of pathogenicity. For example, tissue‐specific molecules such as ENG, vascular endothelial‐growth factor (VEGF) and MMP‐9 are modified in anorectal hemorrhoids (Chung et al., 2004; Lohsiriwat, 2012; Sun and Migaly, 2016); the same molecules that are significantly altered over BAVM‐specific background levels. Although, this working hypothesis is coming into focus, interpreting the functional consequences of any of the above signaling pathways to both BAVMs and anorectal hemorrhoids is challenging. The shared signaling pathways currently listed are broad and should be refined at the level of protein function, cellular signaling and tissue domain to determine whether there is unbiased convergence of ENG, VEGF, and MMPs signatures in both syndromic diseases. Nevertheless, it is clear that both BAVMs and anorectal hemorrhoids show similar arteriovenous pathology, including the clustering of dilated tissue, increased microvascular density and neovascularization correlated with increased VEGF levels (Chung et al., 2004). Thus, a dense and hyperplastic vasculature that results in abnormally aligned endothelial cells in the brain and anorectal plexus has relevance for the etiology of these seemingly distinct conditions (Fig. 5). It should be noted that like BAVMs, the exact pathophysiology of symptomatic anorectal hemorrhoids is poorly understood. It is therefore difficult to know the extent through which specific changes in ENG, VEGF, MMPs, FOXO1, or MEKK3‐KLF2/4 signaling contribute to the burden of these diseases. Unfortunately, research on the interactions between independent and overlapping signaling pathways influencing BAVMs and anorectal hemorrhoids is in its earliest stages. Nevertheless, information gained through parallel investigations of BAVMs and anorectal hemorrhoids would be important for the rational development of therapies aimed to correct common elements of arteriovenous dysfunction.

It should be noted that our working hypothesis aims to establish a new framework for brain research that links common signaling dimensions that may be impaired across multiple disorders. In this regard, there is considerable evidence linking type I interferons to psychiatric disorders (Abbott et al., 1987; Angelino and Treisman, 2005; Baraldi et al., 2012), and the pathophysiology of psoriasis and atherosclerosis may be causally linked as pro‐angiogenic factors including interleukin‐8 (IL‐8), hypoxia inducible factor‐1α (HIF‐1α), protein C‐ETS‐1 (ETS‐1) and VEGF are implicated in the pathogenesis of both diseases (Christophers, 2007; Armstrong et al., 2011; Eder and Gladman, 2015). Moreover, anorectal dysfunction and constipation are well recognized non‐motor symptoms in Parkinson's disease and may reflect the direct involvement of the gastrointestinal tract in response to neuronal damage caused by toxic, aggregation‐prone proteins (Barone, 2010; Cloud and Greene, 2011). Thus, for treatment purposes, it is important to know whether BAVMs are solely diseases of the brain, or whether common signaling dimensions of BAVMs also extend to anorectal structures. This information could provide innovative approaches to the management of BAVMs and potentially mitigate the complications suffered by hemorrhoid‐prone patients.

## Nutritional Substances in the Treatment of Brain Arteriovenous Malformations and Anorectal Hemorrhoids

If future studies do implicate overlapping molecular signaling pathways in common dimensions of BAVMs and anorectal hemorrhoids, are there any therapeutic avenues for reducing some of the symptoms associated with arteriovenous dysfunction? From a therapeutic perspective, one potential option is the use of nutritional substances such as dietary fiber which is thought to help stave off maladies ranging from brain disease to intestinal disorders (Zamroziewicz and Barbey, 2016). Indeed, a low fiber diet is commonly assumed to increase the risk of anorectal hemorrhoids (Peery et al., 2015), and conversely, a fiber‐enriched diet is the primary treatment option as well as a long‐term prophylaxis method for this particular clinical condition (Otles and Ozgoz, 2014). Based upon these observations, can we extrapolate these clinical findings and suggest that consumption of the aforementioned diet might be an effective therapy for BAVMs? Although data still need to be collected, this suggestion may reveal common therapeutics with new potential applications. The onset of age‐related diseases such as BAVMs and symptomatic hemorrhoids could benefit from dietary interventions to reduce inflammation which is also a core feature of pathophysiology for both diseases. Along the same lines, a large body of literature exists to support the view that polyphenols such as resveratrol can activate histone deacetylase pathways (e.g., sirtuins) that mechanistically modulate inflammatory conditions (Baur and Sinclair, 2006; Gan and Mucke, 2008; Baur, 2010; Leheste and Torres, 2015). Thus, there are sufficient clinical data to support the hypothesis that BAVMs and anorectal hemorrhoids may favorably respond to high fiber diet and resveratrol treatment protocols. Clinical trials combining all three phases are worth conducting. In this context, the advantages of using fiber, resveratrol or other nutritional interventions are their safety, broad spectrum utility, low costs and apparent suitability for treating clinical conditions that arise as a function of population history and chance.

An extension of nutritional strategies to improve diseases characterized by abnormal dilated arteries and veins, and defined microscopically by the absence of a capillary network, relates to therapeutic solutions that influence gut microbiotic colonization or metabolism. Indeed, targeting the gut microbiota ecosystem to reduce inflammation, high‐flow nidus and intermitting bleeding as in BAVMs and anorectal hemorrhoids is of tremendous scientific interest. The emerging insights that the brain and intestinal bacteria intersect to shape neurological and alimentary health could especially be relevant to diseases characterized by vascular dysmorphogenesis (Dinan and Cryan, 2013). As the gut microbiota produces thousands of enzymes that specifically target dietary fiber, resveratrol and complex carbohydrates (Bengmark, 2013; Bode et al., 2013; De Vadder et al., 2014; Claes et al., 2015), it is conceivable that dietary interventions that target microorganisms might modulate systemic physiology to reduce the incidence of vessel rupture. Further studies are needed to assess whether manipulation of the gut microbiota is a pragmatic approach to mitigate neurological symptoms that often co‐exist with gastrointestinal distress.

## CONCLUSION

Together, the previous sections of this review provide a snapshot of BAVMs and their clinical manifestations. We raise the possibility that this particular brain disease has several molecular features in common with anorectal hemorrhoids, and propose that consumption of nutritional substances might lower certain risk thresholds associated with arteriovenous malformation disorders. Because the biology underlying the susceptibility to BAVMs is not clear, the following research questions should further be explored: (i) what are the precise underlying mechanisms of the disease? (ii) What is the relationship between the disorder and other more tractable pathologies? (iii) How important is diet for minimizing clinical symptoms? And (iv) can the disorder be identified with specific biomarkers to stratify patients for clinical testing?
